# Self-Assessment of Antimicrobial Stewardship in Primary Care: Self-Reported Practice Using the TARGET Primary Care Self-Assessment Tool

**DOI:** 10.3390/antibiotics6030016

**Published:** 2017-08-16

**Authors:** Rebecca Owens, Leah Ffion Jones, Michael Moore, Dirk Pilat, Cliodna McNulty

**Affiliations:** 1 Public Health England, Gloucester GL50 2QJ, UK; owensfamilyuk@btinternet.com (R.O.); leah.jones@phe.gov.uk (L.F.J.); 2 Department of Primary Care and Population Sciences, Aldermoor Health Centre Southampton, University of Southampton, Southampton SO16 5ST, UK; mvm198@soton.ac.uk; 3 Royal College of General Practitioners, London NW1 2FB, UK; dirk.pilat@nhs.net

**Keywords:** TARGET, antimicrobial resistance, antimicrobial stewardship, primary care, self-assessment, general practice

## Abstract

Multifaceted antimicrobial stewardship (AMS) interventions including: antibiotic guidance, reviews of antibiotic use using audits, education, patient facing materials, and self-assessment, are successful in improving antimicrobial use. We aimed to measure the self-reported AMS activity of staff completing a self-assessment tool (SAT). The Royal College of General Practitioners (RCGP)/Public Health England (PHE) SAT enables participants considering an AMS eLearning course to answer 12 short questions about their AMS activities. Questions cover guidance, audit, and reflection about antibiotic use, patient facing materials, and education. Responses are recorded digitally. Data were collated, anonymised, and exported into Microsoft Excel. Between November 2014 and June 2016, 1415 users completed the SAT. Ninety eight percent reported that they used antibiotic guidance for treating common infections and 63% knew this was available to all prescribers. Ninety four percent of GP respondents reported having used delayed prescribing when appropriate, 25% were not using Read codes, and 62% reported undertaking a practice-wide antibiotic audit in the last two years, of which, 77% developed an audit action plan. Twenty nine percent had undertaken other antibiotic-related clinical courses. Fifty six percent reported sharing patient leaflets covering infection. Many prescribers reported undertaking a range of AMS activities. GP practice managers should ensure that all clinicians have access to prescribing guidance. Antibiotic audits should be encouraged to enable GP staff to understand their prescribing behaviour and address gaps in good practice. Prescribers are not making full use of antibiotic prescribing-related training opportunities. Read coding facilitates more accurate auditing and its use by all clinicians should be encouraged.

## 1. Introduction

The U.K. Antimicrobial Stewardship in Primary Care collaboration came together with the aim of developing an antimicrobial stewardship (AMS) toolkit. The group worked with general practitioners (GPs), educators, and community medicines managers to assess relevant guidelines and develop AMS materials. Within a series of workshops they agreed upon the content of a toolkit to support appropriate antibiotic prescribing [1]. This web-based toolkit, called TARGET Antibiotics, is hosted on the Royal College of General Practitioners (RCGP) website (www.RCGP.org.uk/TARGETantibiotics).

TARGET stands for Treat Antibiotics Responsibly, Guidance, Education, Tools. Many of the materials are based on the Theory of Planned Behaviour [2] and together all can be used to support prescribers’ and patients’ responsible antibiotic use. The resources include an interactive workshop presentation, leaflets to share with patients, audit toolkits, national antibiotic management guidance, training resources, resources for clinical and waiting areas, and a Primary Care Self-Assessment Tool (SAT).

**Figure 1:**
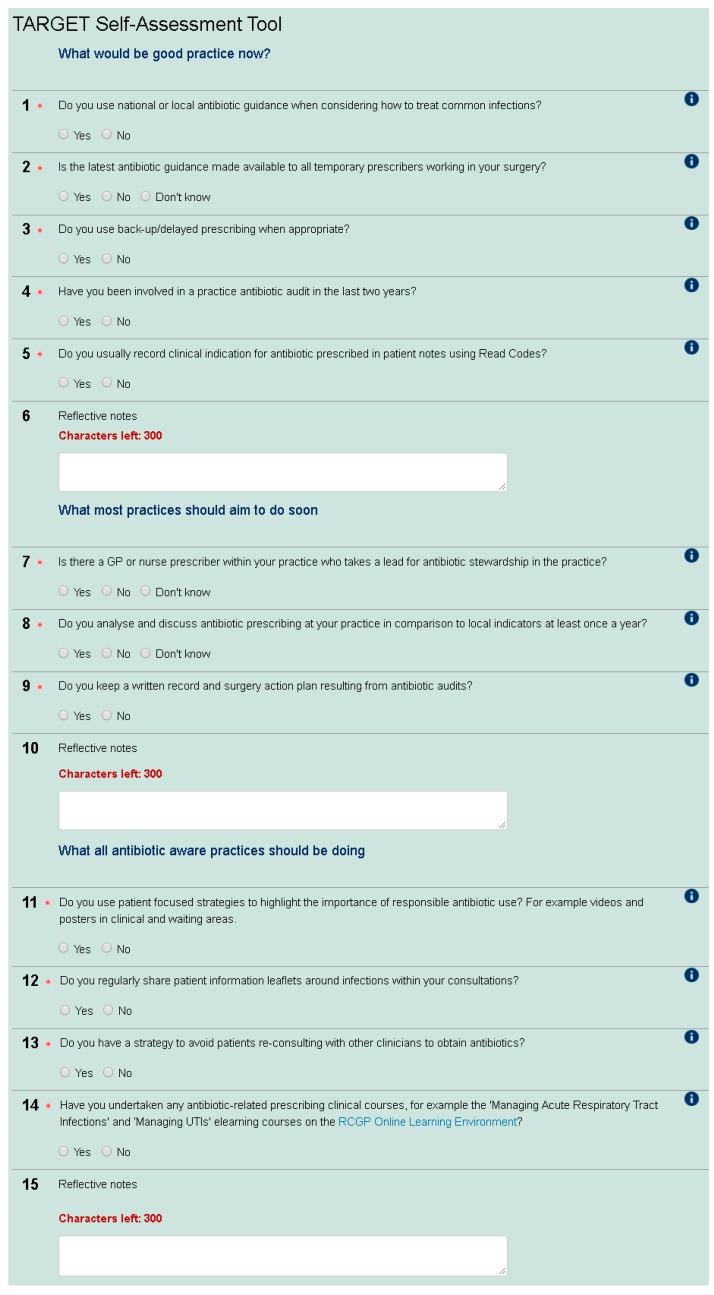
The primary care self-assessment tool.

Based on the Antimicrobial Assessment Toolkit (ASAT) for secondary care [3,4,5], a construct-valid tool developed for secondary care, the primary care SAT enables prescribers to assess their AMS behaviour in comparison to others in their Clinical Commissioning Group (CCG—CCGs are state funded and commission primary health care from general practices in England), nationally and over time. The tool was first developed by The Royal College of General Practitioners (RCGP)/Public Health England (PHE) Antimicrobial Stewardship clinical priority group, which includes GPs, a microbiologist, and pharmacists. The SAT contains 12 yes/no questions around AMS (see Figure 1). This group discussed and agreed what would be good AMS practice now, including antibiotic guidance, audits and using Read codes (a standard set of clinical codes for use in electronic health records), what most practices would aim to do soon (including presence of an AMS lead, evaluation of antibiotic use, and audit action planning), and what all antibiotic aware practices should be doing (including patient focused strategies, patient leaflets, and education undertaken). This wording was used to indicate that all practices should be doing the first group of AMS activities, and most should be moving towards the second group of activities. The SAT also has an educational role as participants can observe the rationale for each question with signposting to materials. The positive wording of “antibiotic aware practices” was used to encourage participants to move in this direction and fulfil the third group of AMS activities. The check list was then tested and refined through user testing with GPs, microbiologists, CCG medicine managers, and pharmacists. The current version was launched in November 2014 and promoted via the RCGP newsletter, the TARGET Antibiotic website, and the RCGP Online Learning Environment (OLE) to any web user. The SAT is available as a non-compulsory precursor to the RCGP online eModule entitled “Antibiotic Resistance in Primary Care” (www.elearning.rcgp.org.uk).

This study aims to understand the AMS activity of primary health care professionals completing the eLearning SAT. Results will be used to inform the future development and promotion of AMS activities and tools by the RCGP and Public Health England (PHE).

## 2. Results

Between November 2014 and June 2016, 2248 users accessed the eModule Antibiotic Resistance in Primary Care that is linked to the SAT. Forty percent of these (897/2248) completed the whole eModule and 63% (1415/2248) completed the SAT. Of these completing the SAT, 92% (1308/1415) went on to complete the module after the SAT. One CPD (Continued professional development) point is available upon completing the module, although it is not compulsory to complete the SAT in order to receive the certificate.

Of those that completed the tool, 91% (1286/1415) were from England, 4% (56/1415) Scotland, 4% (51/1415) Wales and 1% (14/1415) Northern Ireland. Forty eight percent of the English respondents (620/1286) were from the Midlands and 17% (221/1286) were from one CCG in England. Nineteen percent were from the South (excluding London), 19.3% from the North, and 13.1% from London. The SAT was completed by at least 1 person in every health board or CCG, excluding six health boards in Scotland and one CCG in England. The profession of the respondents can be seen in Table 1.

Of those completing the SAT, 96% (1362/1415) were based in general practice, 2% (27/1415) were from an out of hours setting, and the remainder were based either in a hospital, pharmacy, or other workplace. Eighty five percent of respondents (1207/1415) were GPs and 7% (97/1415) were nurses. Two percent (28/1415) were pharmacists. Results are reported as “all respondents” when there are no differences between responders and reported separately when there are differences.

### 2.1. Self-Assessment Tool Sections

#### 2.1.1. What Would Be Good Practice Now

Almost all respondents (98%, 1385/1415) and 100% of nurses reported that they used antibiotic guidance when considering how to treat common infections. Sixty three percent (896/1415) knew that antibiotic guidance was made available to all temporary prescribers in their practice such as locums, trainees, maternity cover, etc. However, nine percent (122/1415) reported that the guidance was not made available to temporary prescribers and a further 28% (397/1415) did not know whether this was the case.

Ninety four percent of GP respondents (1188/1255) and 85% of other respondents (136/160) reported that they used delayed/back-up antibiotic prescribing as appropriate. Seventy five percent of all primary healthcare professionals that completed the tool (1057/1415) said that they recorded clinical indication using Read codes.

**Figure 2:**
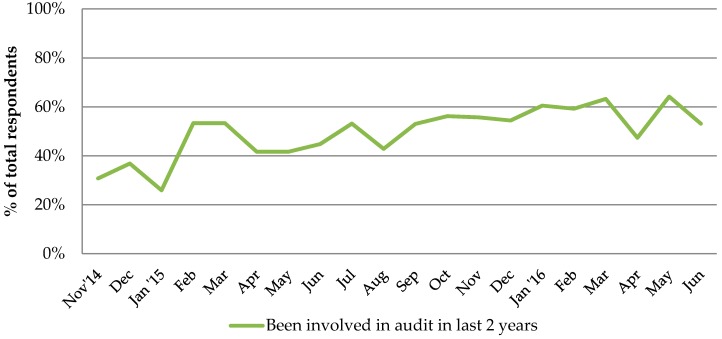
Percentage of respondents involved in an antibiotic audit during last two years.

Overall, less than half of the respondents (45%, 757/1415) reported undertaking a practice-wide antibiotic audit in the last two years. This percentage has increased over time from 30% in November 2014 to 60% in May 2016 (Figure 2). Proportionately, more respondents from the CCG that is highly represented had conducted an audit in the last two years (76%, 169/221) compared to 49% (587/1191) of other respondents from other CCGs. Statistical testing indicates that this is a significant difference (*p* < 0.001).

**Figure 3:**
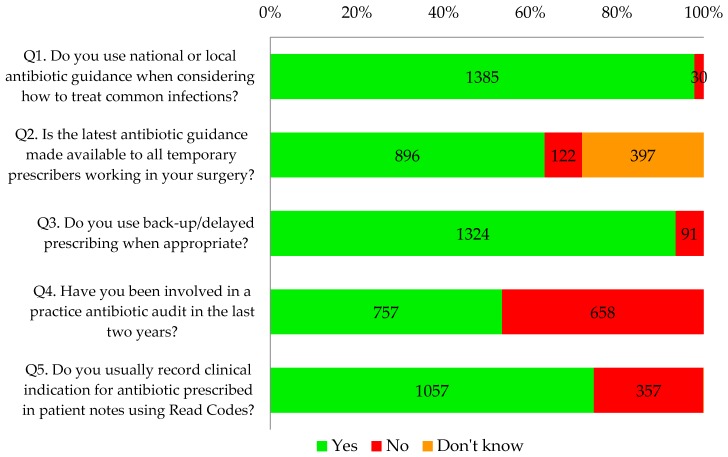
A visual breakdown of the five questions from the section entitled “What would be good practice now”.

Thirty one percent of respondents (432/1415) were carrying out self-reported good practice in antibiotic prescribing as defined by the tool, answering yes to all five questions in this section (Figure 3).

#### 2.1.2. What Most Practices Should Aim to Do Soon

**Figure 4:**
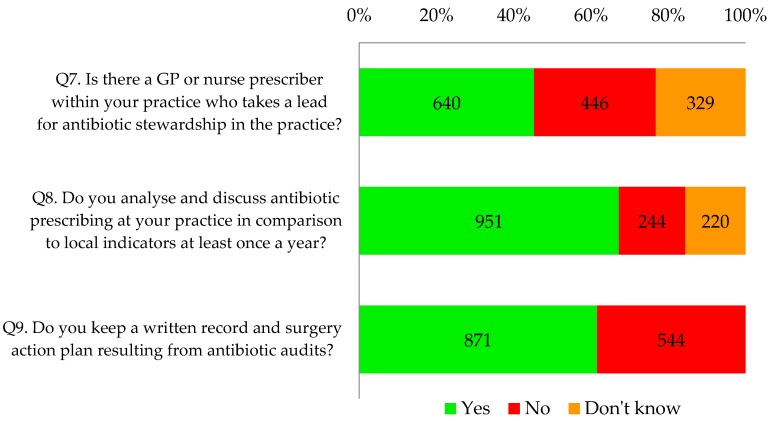
What most practices should aim to do soon.

Thirty two percent of respondents (453/1415) indicated that they or their practice were already fulfilling all the components relating to local leadership of AMS, particularly action plans resulting from antibiotic audits (Figure 4).

Table 2 shows that forty five percent of respondents (640/1415) indicated their GP practice had an identified lead for antimicrobial stewardship. Significantly more respondents from the CCG that was highly represented (78%) indicated that they had an identified antimicrobial stewardship lead (*p* < 0.001). Two thirds of all respondents (67%, 951/1415) reported that they analysed and discussed antibiotic prescribing at least once a year; 62% (871/1415) reported that their practice kept a written record and action plan resulting from antibiotic audits.

Of those that reported undertaking a practice-wide antibiotic audit in the last two years, 84% (639/757) also said that they analysed and discussed antibiotic prescribing at their practice in comparison to local indicators at least once a year. Seventy seven percent (584/757) also kept a written surgery action plan resulting from audits.

#### 2.1.3. What All Antibiotic Aware Practices Should Be Doing

**Figure 5:**
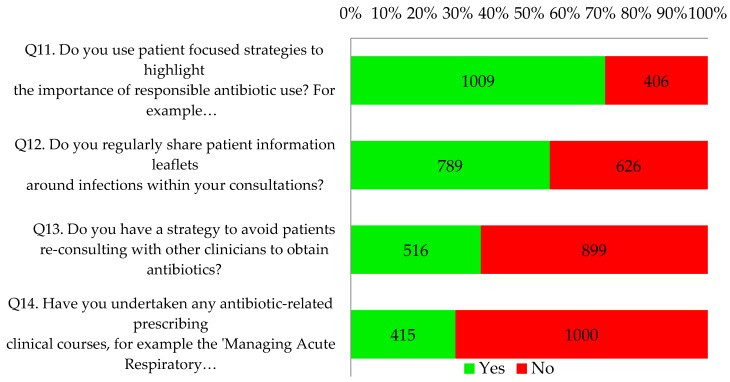
What all antibiotic aware practices should be doing.

Only 8% of respondents reported that their practices (110/1415) were already fulfilling the four key criteria of an antibiotic aware practice (Figure 5). Twenty five percent of respondents (348/1415) answered yes to three questions.

Seventy one percent of respondents (1009/1415) reported using patient focused strategies to highlight the importance of responsible antibiotic use, such as videos and posters in clinical and waiting areas. Just over half of respondents (56%, 789/1415) indicated that they regularly shared patient information leaflets around infections within consultations. Proportionately more nurse respondents indicated that they did this (84%, 86/102; *p* < 0.001 vs. 54% of the other professionals). Two thirds of all respondents had a practice strategy to avoid patients re-consulting to obtain antibiotics. Most (71%, 1000/1415) had not undertaken any antibiotic-related prescribing clinical courses but the reflective notes did suggest that many intended to undertake further online training as part of their personal development plan. This is illustrated by the following quotes:

Need to do the ELearning course and to share with practice staff.

—GP 1281, Wigan

Need to improve my education, colleagues’ education and patients.

—GP 1150, Belfast

## 3. Discussion

### 3.1. Summary

Our analysis suggests that many prescribers using the SAT are carrying out self-reported “good practice” regarding antimicrobial stewardship, as almost all are using antibiotic guidance to assist prescribing decisions. Many are also using delayed prescribing when they consider it appropriate and are making use of Read codes to record clinical indication. While it is positive to see that many are using antibiotic guidance to assist prescribing decision making, it is disappointing that only 63% knew that the guidance was made available to temporary prescribers in their clinical setting, nine percent said that it was not, and a further 28% didn’t know. It is important that antibiotic management guidance is made available to all prescribers within a practice, either by signposting to online sources or by having printed versions within each consultation room. This ensures that a consistent approach is used across a practice and minimises the chance of a patient reinforcing consulting behaviour by reconsulting with and obtaining antibiotics from another clinician within the practice.

Our findings suggest that there is scope for improvement in planning and monitoring AMS and associated prescribing activity at a GP practice level. Twenty five percent of participants were not using Read coding that facilitates audits and not all practices are maximising the use of auditing and action planning to understand and address local antibiotic prescribing. Less than half (45%) had an identified practice lead for stewardship, although just over two thirds (67%) analysed and discussed antibiotic prescribing in comparison to local indicators at least once a year. The least popular stewardship activity was participation in antibiotic-related prescribing clinical courses, with less than a third reporting to having done this.

We have noted some positive examples of sharing information with patients: over 70% of respondents are using posters and videos to highlight the importance of responsible antibiotic use. However, only just over one half of respondents regularly shared any kind of patient information leaflets; it is not known which leaflets were shared.

### 3.2. Strengths and Limitations

A key strength of this work is the large sample of 1415 GP staff from CCGs and health boards across the U.K., over the course of 18 months and providing a self-reported view of both individual and practice AMS activity. However, this is also a limitation as this represents around 2% of the total GP population in the U.K. [6] and the SAT doesn’t allow respondents to distinguish between whether they are in training or not, or whether they are prescribers or not. Additionally, the respondents have not been selected randomly nor systematically, therefore may represent a keen section of GP staff, nor do we know of specific local audits, promotions, or requirements which may have influenced responses over time. However, in appraisals, GPs must show evidence of quality improvement activities. Our findings indicate a room for improvement in areas of AMS (other than the use of guidance), even in this group.

As respondents completed an anonymised form they were not influenced by their team or by an interviewer and therefore were free to record activity in a reflective manner. It is therefore unlikely that respondents were influenced by observer bias and were more likely to provide an accurate reflection of their AMS. Conversely, this makes it difficult to analyse the responses in relation to objective measures such as prescribing rates. It is also impossible to provide follow up questions to respondents, e.g., to find out more about their reported AMS activities.

The proportion reporting they use delayed/back-up prescribing when appropriate was high, at 94% of GPs. This high percentage could also be because we had an “antibiotic interested” sample, or that the term “appropriate” was interpreted differently by respondents. In another U.K. study [7], only half of participating practices returned data with delayed/back-up prescribing and overall approximately 25% of delayed/back-up antibiotic prescriptions were actually delayed.

The wording of the questions within the SAT does not enable us to explore exact detail of AMS activities within each section. For example, the question about delayed prescribing doesn’t ask when they used this strategy or how, and therefore is limiting. It would be useful to add open questions about the different AMS strategies used. Similarly, we only have limited information on the Read coding activity. We do not know, for example, the quality of the Read coding that is conducted.

In this sample, 77% of those that had conducted an antibiotic audit had developed a surgery action plan as a result of the audit. It has been argued that audits are not effective at changing behaviour if the audits are not complete, if re-auditing is not carried out, or if the audits do not lead to a firm action plan with the aim of quality improvement [8]. In this context, the SAT facilitates the examination of re-auditing and action planning. However, perhaps the SAT would be improved if it enabled further investigation of audit activity by highlighting to primary care clinicians when their auditing has been effective or ineffective at changing behaviour.

### 3.3. Comparison with Existing Literature

A 2014/15 survey of CCGs exploring local implementation of national AMS toolkits found that 84% of responding CCGs had implemented AMS antibiotic audits in GP practices [9]. However, this survey incurred a relatively low response rate of 39% of CCGs. In contrast, only 53% of GP staff in our study reported conducting an audit in the last two years, indicating a mismatch between CCG perceptions and actual audits undertaken by GP staff at practice level. This indicates that questioning CCGs may not give a true reflection of activities at the GP practice level.

A Patients Association report released in 2016 [10] reported on CCG AMS activity and explored local implementation of the TARGET Antibiotics Toolkit. Although these findings also relate to CCG rather than individual prescriber or GP practice AMS activity, it is nevertheless interesting to note that 48% reported implementing audits locally and 69% reported they had a named individual responsible for the implementation of AMS. This compares with 54% and 69% respectively in our study. Forty percent of respondents in the Patient Association study had accessed the training resources on the TARGET Antibiotics Toolkit which contrasts again with the 32% in our study that had undertaken antibiotic-related prescribing clinical courses. We consider that our results are probably a more realistic assessment of practice based audit and training activities.

In 2016, almost three quarters of GPs were familiar with the NICE (National Institute for Health and Care Excellence) Antimicrobial Stewardship Guideline [11,12] and a number of its recommendations had been carried out locally. It concluded that more needed to be done locally around patient education of AMR. Our study concurs with this finding but also shows there needs to be further GP education around AMR and the promotion and use of tools that are available to support better prescribing.

### 3.4. Implications for Research and Practice

#### 3.4.1. Implications for GP Practices

The sample used in this study is arguably a favourable representation of AMS activity, therefore the reality of nationwide stewardship activities is likely to be less than reported here. Despite this, the findings highlight important areas for improvement in this sample and beyond. This study shows that antibiotic guidance, the bedrock of AMS, is being widely implemented. However, GP practice managers need to ensure that temporary prescribers have access to this antibiotic prescribing guidance. A quarter of our respondents were not using Read codes and nearly half had not conducted an antibiotic audit. These should be encouraged as they are an effective means of enabling GP staff to understand their prescribing behaviour and address gaps in good practice. Some respondents (17%) had not compared their own prescribing with local indicators. This is now much easier to do and should be encouraged using data on websites such as PrescQIPP [13], Fingertips [14], or OpenPrescribing [15].

Recent qualitative research relating to the TARGET resources [16] highlights a need for easy access to materials and a lack of knowledge about audits. These findings were replicated in our study and there is scope for implementing further patient-focused strategies such as providing leaflets, videos and posters to discourage re-consultation, and improve patient understanding of self-care.

Prescribers are not making full use of the antibiotic-related prescribing training opportunities that are available online through the TARGET Antibiotics Toolkit and elsewhere, and may need more dedicated time or incentives to be able to do this.

#### 3.4.2. Implications for CCGs

The Quality Premium (QP) is a financial incentive intended to reward CCGs for quality improvement. The QP for 2017/18 contains an antibiotic component to reduce total antibiotic prescribing by 1% and broad-spectrum antibiotics by 10%. CCGs (and potentially GP practices) can be rewarded financially for improvement in this respect and it has therefore generated much interest and subsequent AMS activity. Our study highlights an opportunity for CCGs to provide support for regular and appropriate audits, therefore monitoring progress in meeting QP targets.

As we have indicated, there is a need for GPs to conduct further antibiotic related training. CCGs should encourage this and use other supporting resources in the TARGET Antibiotics Toolkit. Indeed some are doing so through enhanced payment incentive schemes.

#### 3.4.3. Implications for Research

It is important to explore in more depth the issues raised by this study, especially now that further antimicrobial stewardship initiatives have been introduced in the UK. Our analysis suggests that there is some good activity underway nationally and further research using an objective methodology is necessary to identify activities within individual CCGs, the extent of activity implementation and adoption, and the overall success of activities in terms of resistance rates and antimicrobial prescribing.

PHE promotes TARGET via events and RCGP communications, but rely on CCGs and local teams to promote the resources locally. As we have highlighted, there is a particularly high number of responses in our study from one CCG. Further investigation suggests that this CCG circulates regular newsletters containing information about TARGET. However, we do not know to what extent this is enough to generate a higher level of interest in the SAT nor do we yet have a comprehensive picture of what other CCGs are doing in this respect. Further research examining the exact nature of AMS activities in more detail would also address these issues.

## 4. Materials and Methods

The responses to the SAT are recorded digitally on the RCGP server and participants can print their responses and CPD certificate on completion. Data were collated by RCGP Learning between November 2014 and June 2016, anonymized, and exported into Microsoft Excel.

Data was cleaned to remove duplicate participants, subsequent attempts, or entries in which no questions were completed. It was then analysed using Microsoft Excel and comparisons were made between different respondents. Chi-squared testing was used to determine if participant type influenced the responses of GPs and “others” (including nurses, pharmacists and others). Further testing was carried out for each question on the disproportionately high number of responses that came from a specific CCG to see whether there were significant differences from the main cohort. Any significant differences are reported in our results.

Ethical approval was not required for this piece of work as it was a service evaluation and the involved staff and all data obtained was anonymised by the RCGP by removing email addresses and any identifiable information.

## 5. Conclusions

Our findings suggest that there is scope for improvement in planning and monitoring AMS and associated prescribing activity at a GP practice level. Respondents to the SAT reported undertaking a variety of AMS activities to differing extents. It is important to ensure that all clinicians have access to prescribing guidance to facilitate accurate prescribing. The use of antibiotic audits should be reinforced to enable clinicians to understand their prescribing behaviour, address gaps in good practice and create effective action plans. Prescribers are not making full use of antibiotic prescribing-related training opportunities. Read coding facilitates more accurate auditing and its use by all clinicians should be encouraged.

## Figures and Tables

**Table 1:** Profession of respondents.

Professional Group	Number	Percent (%)
GP	1255	88.7
Nurse	102	7.2
Pharmacist	28	2.0

**Table 2:** Audit and action planning.

	Number of “Yes“ Answers	Percentage of Total Answering Yes (%)
Has been involved in antibiotic audit in last 2 years	757	53.5
Has a practice lead for antibiotic stewardship	640	45.2
Discusses antibiotic prescribing within the practice compared with local indicators	951	67.2
Keeps a written record and surgery action plan resulting from audits	871	61.6
